# A systems biology approach to investigate the mechanism of action of trabectedin in a model of myelomonocytic leukemia

**DOI:** 10.1038/tpj.2016.76

**Published:** 2016-12-13

**Authors:** L Mannarino, L Paracchini, I Craparotta, M Romano, S Marchini, R Gatta, E Erba, L Clivio, C Romualdi, M D’Incalci, L Beltrame, L Pattini

**Affiliations:** 1 Department of Oncology, IRCCS-Istituto di Ricerche Farmacologiche Mario Negri, Milan, Italy; 2 Department of Bioscience, University of Milan, Milan, Italy; 3 Department of Biology, University of Padua, Padua, Italy; 4 Department of Electronics, Information and Bioengineering, Politecnico di Milano, Milan, Italy

## Abstract

This study was designed to investigate the mode of action of trabectedin in myelomonocytic leukemia cells by applying systems biology approaches to mine gene expression profiling data and pharmacological assessment of the cellular effects. Significant enrichment was found in regulons of target genes inferred for specific transcription factors, among which *MAFB* was the most upregulated after treatment and was central in the transcriptional network likely to be relevant for the specific therapeutic effects of trabectedin against myelomonocytic cells. Using the Connectivity Map, similarity among transcriptional signatures elicited by treatment with different compounds was investigated, showing a high degree of similarity between transcriptional signatures of trabectedin and those of the topoisomerase I inhibitor, irinotecan, and an anti-dopaminergic antagonist, thioridazine. The study highlights the potential importance of systems biology approaches to generate new hypotheses that are experimentally testable to define the specificity of the mechanism of action of drugs.

## Introduction

Trabectedin (ET-743) is a DNA minor-groove binding agent originally isolated from the Caribbean tunicate *Ecteinascidia turbinata* and recently obtained by chemical synthesis.^1^ It is approved for the second line therapy of soft-tissue sarcoma and ovarian cancer in Europe, and several other countries and is currently under evaluation in many other malignancies. It has a complex mode of action involving DNA repair and transcription mechanisms, resulting in cytotoxic effects with perturbations of the cell cycle and cell death (D'Incalci *et al.*^2^, for a review). Previous studies have indicated that trabectedin is cytotoxic not only against cancer cells, but exerts also a selective activity against monocytes and macrophages.^3, 4^ This finding was of interest as macrophages are quiescent cells generally considered very resistant to most anticancer drugs. These observations led to discover that trabectedin has the ability to modulate the tumor microenvironment,^5^ that was proposed to be relevant also for its antitumor activity.

Recently, the drug has shown a significant cytotoxic activity against Juvenile and Chronic myelomonocytic cells taken from patients suffering from these diseases for which there is an urgent need of novel effective therapies.^6^ This observation prompted us to investigate the mode of action of trabectedin in a representative myelomonocytic leukemia cell line. We have applied a systems biology approach to study gene expression profiles of treated cells integrated with specific experiments aimed at assessing their phenotype. The results indicate that the drug has the ability to modulate specific pathways regulating cell cycle and cell death of myelomonocyctic leukemic cells, thus reinforcing the idea that the drug has potential therapeutic action against myelomonocytic leukemias.

## Materials and methods

### Drugs

Trabectedin was supplied by PharmaMar (Colmenar Viejo, Madrid, Spain) and was stocked in dimethyl sulfoxide at a concentration of 1 mM and stored at −20 °C. 5-azacytidine, thioridazine and SN-38 were purchased from Sigma (St Louis, MO, USA). All drugs were diluted in medium just before use.

### Cell growth and treatment

MV-4-11, a biphenotypic B myelomonocytic leukemia, cell line was grown in RPMI-1640 medium (Life Technologies,Carlsbad, CA, USA) supplemented with 10% of fetal bovine serum HyClone (Thermo Scientific, Waltham, MA, USA) and 1% L-glutamine 200 nM (Life Technologies, Milan, Italy). Cells were maintained at 37 **°**C and in a humidified atmosphere at 5% CO_2_ in 25 cm^2^ flasks (Iwaky Bibby Sterilin, Staffordshire, UK). Exponentially growing cells were treated with different concentrations of either trabectedin or 5-azacytidine, or thioridazine, or SN-38. Cell growth inhibition was measured by counting the number of cells by using Coulter Counter (Beckman Coulter, Brea, CA, USA).

### Cell cycle perturbation and apoptotic cell death by flow cytometry

To evaluate the cell cycle perturbation, treated and untreated cells were fixed in ethanol 70%, washed in cold phosphate-buffered saline and the DNA was stained overnight with 1 ml of a solution containing 12,5 g ml^−1^ propidium iodide and 12,5 μl of RNAse 1 mg ml^−1^. To detect apoptosis, MV-4-11 control and treated cells were processed by using the Annexin-V detection kit FITC (Affymetrix eBioscience, St Clara, CA, USA), following the manufacturer’s protocol. Flow cytometric analysis was performed on 10 000 events by using FACS Calibur instrument (Becton Dickinson, Franklin Lakes, NJ, USA).

### Microarray experiment and data quantification

RNA was extracted using a commercial kit (miRNeasy, QIAGEN, Milan, Italy), according to the manufacturer's instructions. 100 ng of total RNA were reverse-transcribed into Cy3-labeled cRNA using LowInput QuickAmp labeling kit (Agilent Technologies, Palo Alto, CA, USA), and hybridized onto commercially available array platforms as previously described.^7^ Three technical replicates were used for each condition. Raw data from Agilent Feature Extraction version 11 were preprocessed removing features marked as unreliable by the scanning software and arrays were normalized using the ‘quantile’ method.^8^ Raw data are available on the ArrayExpress database, under accession ID E-MTAB-2978.

### Differential expression analysis

The R package limma (Linear models for microarray analysis)^9^ was used to determine differentially expressed genes (DEGs) applying a correction for technical replicates. A time-course analysis was accomplished: DEGs both for trabectedin and 5-azacytidine treatment were calculated by comparing 6 h versus control and 24 h versus 6 h samples. Untreated samples were used as control. Genes with a false discovery rate (FDR)^10^ corrected *P*-value (*q*-value) ⩽0.01 were deemed significant.

### Functional annotation analysis

Pathway analysis was performed using a topology-based approach,^11^ with Reactome^12^ and the Pathway Interaction Database (PID^13^) as annotation sources. Pathways were deemed significant if their *q*-value was ⩽0.05.

Gene Ontology functional enrichment was performed by querying the Gene Ontology source^14^ using the biological process ontology on both upregulated and downregulated genes differentially expressed by both the treatment with trabectedin and the drugs resulted from CMAP analysis. Nominal *P*-values were corrected with the Bonferroni method (corrected *P*-values⩽0.01).

### Master regulator analysis

A master regulator analysis was applied to infer the transcription factors mainly involved in specific deregulation of gene expression of drug responding cells. To this aim, a transcriptional network was reverse engineered from a publicly available clinical database of expression profiles of 22215 genes related to 293 patients affected with acute myeloid leukemia (NCBI Gene Expression Omnibus GSE1159) starting from a list of all known human transcription factors.^15^ The ensemble of transcriptionally correlated genes (regulon) for each transcription factor was estimated through the ARACNE software,^16^ based on the mutual information operator. For each regulon, only positively (sign assessed according to Pearson’s correlation coefficient) correlated targets were considered and gene sets with at least 20 genes were retained. The regulons obtained were used for Gene Set Enrichment Analysis^17^ comparing gene expression data from either trabectedin or 5-azacytidine treatments at 6 and 24 h with control samples (0 h) independently, with default parameters.

### Search for similar drug profiles

We queried the Connectivity MAP (CMAP^18^) to search for drug responses with gene expression profiles similar to trabectedin response. Up- and downregulated genes related to the treatment with trabectedin at 6 h were used as input, converting Agilent probes identifiers in Affymetrix GeneChip Human Genome U133A Array identifiers using the conversion ID tool provided by DAVID.^19^

## Results and Discussion

**Figure 1:**
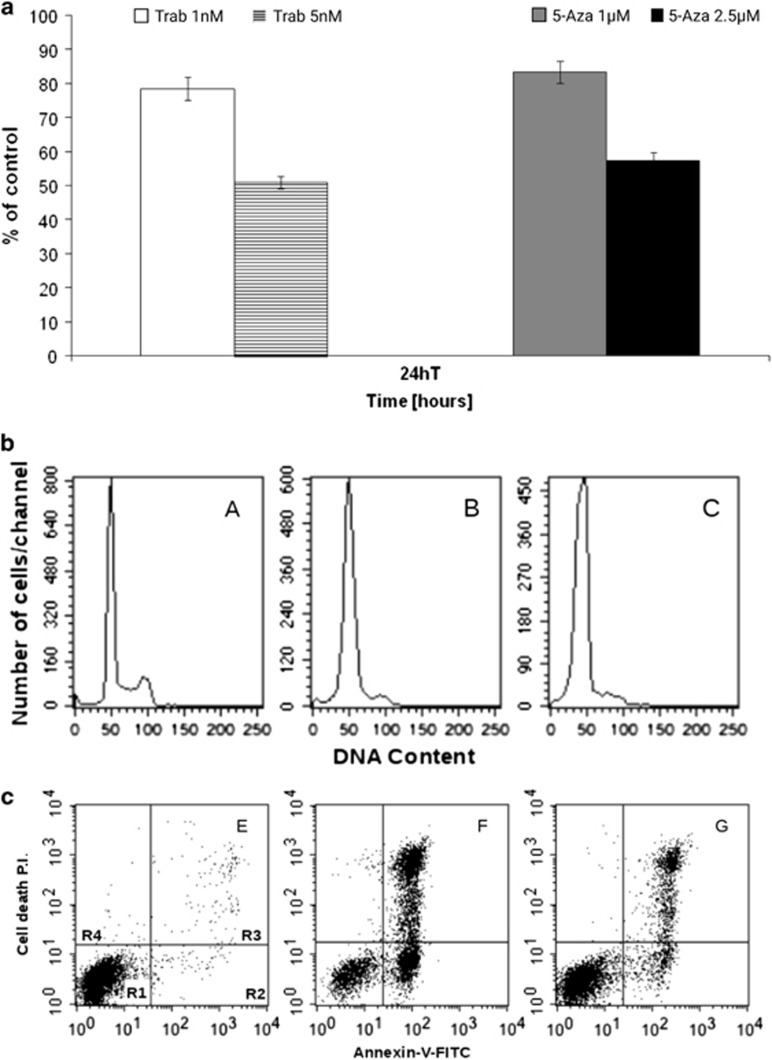
(**a**) Growth inhibition induced by different concentrations of trabectedin (Trab) or 5-azacytidine (5-Aza). 5 nM and 2.5 μM correspond to the half-maximal inhibitory concentrations (IC_50_) for trabectedin and 5-azacytidine, respectively. (**b**) Cell cycle perturbation induced by 5 nM trabectedin (B) and 2.5 μM 5-azacytidine (C) evaluated at 24 h after drug-treatment compared to the control cells (A). (**c**) Apoptosis induced by 5 nM trabectedin (F) and 2.5 μM 5-azacytidine (G) evaluated at 24 h after drug-treatment compared with the control cells (E). P.I., propidium iodide. R1, viable cells; R2, early apoptosis; R3, late apoptosis; R4, necrosis.

**Figure 2:**
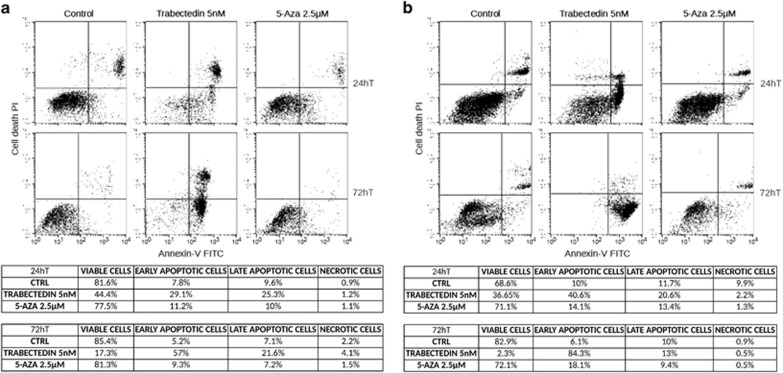
Apoptosis induced by 5 nM trabectedin and 2.5 μM 5-azacytidine (5-AZA) evaluated at 24 and 72 h after drug-treatment on cells derived from either a JMML patient (**a**) or a CMML patient (**b**). Tables report the percentage of apoptotic and viable cells as average of three different JMML (**a**) or CMML (**b**) patients at 24 and 72 h after treatment. R1: viable cells; R2: early apoptosis; R3: late apoptosis; R4: necrosis.

In the present study, we show that trabectedin is very effective in inhibiting cell proliferation and inducing apoptosis in MV-4-11 cells. Figure 1a shows that a significant growth inhibition was achieved with 5 nM trabectedin or 2.5 μM 5-azacytidine. Figure 1b reports the FACS analysis of cell cycle distribution of MV-4-11 cells exposed to the equiactive concentration of trabectedin and 5-azacytidine for 24 h. Both drugs caused a delay in crossing G1 through S phase of the cell cycle. AnnexinV/PI flow cytometric assay revealed that trabectedin induced a remarkable apoptosis, as the fraction of apoptotic cells evaluated at 24 h of treatment was 70%, whereas this fraction was only 30% after 5-azacytidine treatment (Figure 1c). These data were then confirmed on other cells representative of human myelomonocytic leukemia. We obtained fresh leukemia cells from patients suffering from JMML or CMML, respectively. In Figure 2, the induction of apoptosis by 5 nM trabectedin treatment is shown in representative JMML (Figure 2a) and CMML (Figure 2b) primary cultures. The effect was much greater than that of 2.5 μM 5-azacytidine used as positive control, confirming results obtained on the MV-4-11 cell line.

Altogether, these data confirmed the high potency of trabectedin on myelomonocytic leukemia cells, thus justifying studies to elucidate its molecular mechanism.

**Figure 3:**
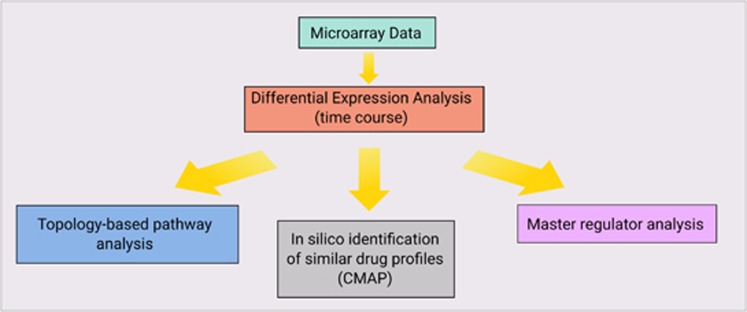
Workflow of the study.

**Figure 4:**
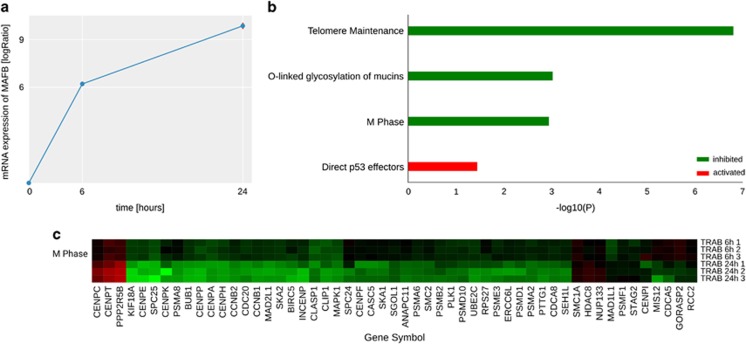
(**a**) Expression trend of the *MAFB* log2 ratio versus the reference (time point 0) from 6 to 24 h. (**b**) Pathways related to deregulated genes with trabectedin treatment. FDR *q*-values are shown in logaritmic scale (base 10). (**c**) M-phase heatmap showing DEGs of trabectedin at both 6 and 24 h annotated with the Reactome database.^12^

Gene expression analysis was performed to investigate early changes in transcriptional regulation induced by trabectedin or 5-azacytidine after 6 and 24 h of treatment (Supplementary Tables 1-2), according to the workflow reported in Figure 3. Differential analysis revealed that *MAFB* (fold change 9.10) was the most upregulated gene at 24 h (Figure 4a). We performed a pathway analysis of deregulated genes identifying pathways relevant to response and their activation state (Materials and Methods). Among our results related to trabectedin, we found the inhibition of the pathway related to M-phase at 24 h (Figure 4b and c).

As it is reported that one of the main mechanisms of action of trabectedin is the modulation of transcriptional regulators, we focused our attention on transcription factors using an approach based on master regulator analysis.

Results reported in Supplementary Table 3 show 12 significant (FDR-corrected *P*-value<0.05) regulons at 6 h and 50 regulons at 24 h, both at FDR<5%.^10^ A number of transcription factors such as *JUNB*, *OVOL2*, *NR4A2*, *NR4A1*, *NFKB2*, *ID2* and *ZBTB7B* were found to be enriched both at 6 h and at 24 h.

**Figure 5:**
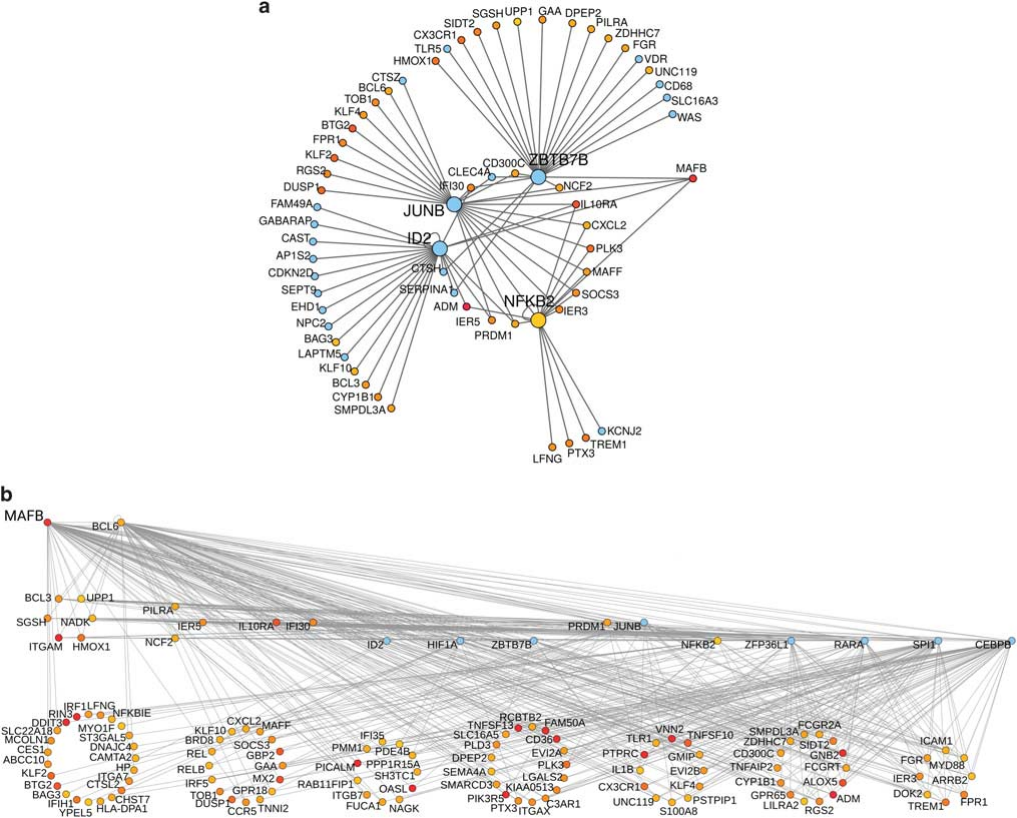
*MAFB* networks. Enriched network at 6 h: *ZBTB7B*, *JUNB*, *NFKB2* and *ID2* hub genes; *MAFB* interacts in their network (**a**). Enriched network at 24 h: *MAFB* is the most interconnected hub (**b**). Light blue, genes of the enriched network of Gene Set Enrichment Analysis (GSEA) but not differentially expressed; from yellow to red, upregulated DEGs, the darker the color, the stronger the upregulation.

The most upregulated gene at 24 h, *MAFB*, seemed to have a crucial role in the transcriptional network. First, at 6 h it was present in the area of influence of four enriched transcription factors, such as *JUNB*, *ID2*, *ZBTB7B* and *NFKB2*, (Figure 5a). Then, at 24 h *MAFB* itself came up to be one of the most enriched transcription factors, and at the same time it was also the highest linked element of the network. Indeed, the most significant genes in the enrichment analysis of *MAFB* regulon showed a coherent behavior in the expression values trend (Supplementary Figure 1). *MAFB* belongs to the Maf leucine zipper transcription factor family, which is expressed specifically in myeloid cells, where its upregulation is correlated to monocyte differentiation for which it plays a crucial role.^20^ Further studies by Gemelli and coworkers demonstrated that *MAFB* is a master regulator of human monocytopoiesis.^21^ Moreover, among these enriched transcription factors, we found *JUNB* and *FOSL2*, whose protein product is known to dimerize in the AP-1 complex transcription factor, already described for its capacity to regulate monocyte differentiation.^21^ Interestingly, Gemelli *et al.* investigated also the genetic program activated by *MAFB* transduction and found that the AP-1 complex transcription factor was directly upregulated by *MAFB*.^21^ In order to assess the specificity of our results, we compared the enrichment results of trabectedin at 24 h versus control and of 5-azacytidine versus control at the same time point. We found 14 transcription factors related only to trabectedin, among which *MAFB*, and not to 5-azacytidine (Supplementary Table 4).

**Figure 6:**
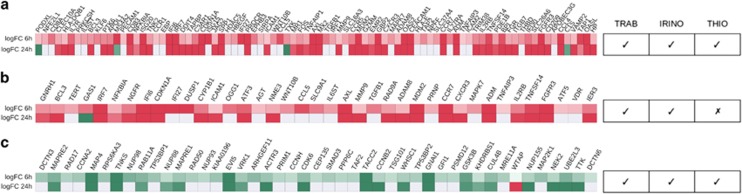
Upregulated genes at 6 h shared between trabectedin and irinotecan and between trabectedin and thioridazine related to the immune system process (**a**). Upregulated genes at 6h in common between trabectedin and irinotecan involved in to apoptotic process (**b**). Downregulated genes at the same time shared by the three drugs associated to the cell cycle network (**c**). logFC, log2-transformed fold change between treatment and control. Tick marks identify the presence of the gene signature for a specific drug, cross marks indicate absence.

We then searched for a gene profile-based similarity of MV-4-11 cells treated with trabectedin with other known compounds, using the Connectivity MAP.^18^ Irinotecan was the highest ranked compound, before phenoxybenzamine, resveratrol and thioridazine (Table 1). First, we focused our attention on irinotecan, the highest ranked result, and thioridazine, because of the major number of gene signatures correlated with our query (Table 1). The first drug, irinotecan, is a camptothecin analog developed as anticancer in the early 1970s.^22^ As for all camptothecins, the mechanism of action of irinotecan consists in the binding of DNA-topisomerase I, causing the formation of a ternary complex with DNA that is then converted in DNA breaks. The other correlated compound, thioridazine, is a phenothiazine, acting as antagonist of the dopamine receptor D2 family proteins.^23^ Although commonly used as antipsychotic drug,^23^ previous studies have revealed its anticancer action.^23, 24^ The positive correlation between trabectedin and these two drugs led us to think about possible similarities between their mechanism of action. For this reason, we performed two independent Gene Ontology analyses on the gene signatures in common between trabectedin and irinotecan and trabectedin and thioridazine, respectively. We found that upregulated genes shared by both comparisons were associated to the immune system, whereas downregulated genes were related to the cell cycle process (Figure 6a and c, respectively). Moreover, upregulated genes shared by trabectedin and irinotecan were also associated with apoptosis (Figure 6b). In order to confirm these findings, we tested with qRT-PCR four upregulated genes shared by trabectedin, irinotecan and thioridazine, but not differentially expressed with 5-azacytidine, that is *CCR7*, *GBP2*, *MDM2* and *PROCR* (Supplementary Figure 2). The combination of trabectedin with irinotecan seems very interesting since it has been recently found effective in preclinical models and very recently there has been a preliminary indication of its clinical efficacy.^25, 26^ The two drugs were used in a context of synergistic action and molecularly targeted combination therapy, since trabectedin blocks the *EWS-FLI1* fusion gene activity, thus making sarcoma cells more sensitive to irinotecan.^25, 27^ On the other hand, it has recently been demonstrated that thioridazine selectively targets and impairs somatic cancer stem cells capable of *in vivo* leukemic disease, while sparing normal cells.^23^ This drug also suppresses proliferation and induces apoptosis in leukemic cells, and it is the most effective compound among the other phenothiazines.^28^ Moreover, Min *et al.*^29^ stated that thioridazine stimulates TRAIL-mediated apoptosis in various human carcinoma cells and this mechanism is also related to the action of trabectedin on tumor-associated macrophages.^5^ These data have prompted a clinical trial on the combination of thioridazine and cytarabine for the treatment of relapsed or refractory Acute Myeloid Leukemia (https://clinicaltrials.gov/show/NCT02096289).

From our analysis we also found a positive correlation with phenoxybenzamine and resveratrol. While not the subject of our study, both drugs have interesting features. The first is a noncompetitive antagonist of *α*_1_- and *α*_2_-adrenergic receptors,^30^ whereas the second is a phytoalexin derived from the skin of grapes and other fruits, which is known to have anti-inflammatory and anti-oxidant effects,^31^ as well as being a chemopreventive agent.^32^ Moreover, it was demonstrated that resveratrol has the capacity to induce cell death in leukemia cells, in particular acute myeloid leukemia cells, sensitizing them to the action of the histone deacetylase inhibitors (HDACIs).^33^

Interestingly, among the biological functions that were shared by irinotecan, thioridazine and trabectedin, such as immune system process, immune response and apoptosis, we found the involvement of the p53 effector *CDKN1A* gene. This gene, also known as p21, is a cyclin-dependent kinase inhibitor, mainly regulated at transcriptional level.^34^ Its main function is to negatively modulate cell cycle progression and block it over the G1-S phases.^34^ Our flow cytometry experiments confirmed the inhibition of cell cycle exactly in these phases. Moreover, as previously reported,^34^ *CDKN1A* is upregulated during hematopoietic differentiation, and this upregulation increases as cells differentiate.^35^ In the same review, Steinman at al. confirmed that the upregulation on p21 in hematopoietic precursor cells leads to the differentiation towards the myelomonocytic lineage. In our study, *CDKN1A* resulted strongly upregulated at both 6 and 24 h (log-fold change of nearly 10 and 15, respectively), and further confirmed also by qRT-PCR as shown in Supplementary Figure 3. In the same experiment we also noticed that *CDKN1A* was not significantly expressed by 5-azacytidine treatment at both time points. To further assess the specificity of *CDKN1A*, we performed the same Gene Ontology analysis done on irinotecan and thioridazine signatures using 5-azacytidine signatures. Only the immune system process was shared between the two drugs signatures. Therefore, this evidence indicates that the mechanisms we observed in this cell line may be trabectedin specific.

**Figure 7:**
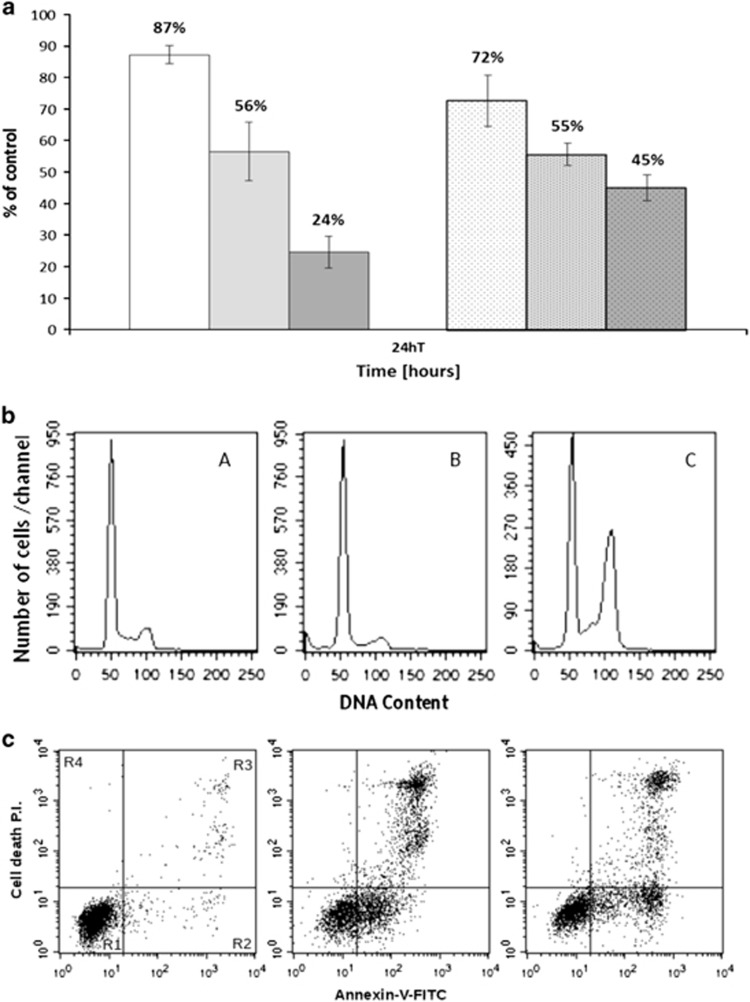
(**a**) Growth inhibition induced by different concentrations of thioridazine or SN-38. 10 μM and 5 nM represent the half-maximal inhibitory (IC_50_) concentrations for thioridazine and SN-38, respectively. (**b**) Cell cycle perturbation induced by 10 μM thioridazide (B) and 5 nM SN38 (C) evaluated at 24 h after drug-treatment compared to the control cells (A). (**c**) Apoptosis induced by 10 μM thioridazine (F) and 5 nM SN38 (G) evaluated at 24 h after drug-treatment compared to the control cells (E). P.I., Propidium Iodide; R1, viable cells; R2, early apoptosis; R3, late apoptosis; R4, necrosis.

Considering the similarities between the transcriptional effects of trabectedin and those of camptothecin or thioridazine, we wondered whether the drugs had also a similar effect on cell cycle and apoptosis. We found that both thioridazine and SN-38, the active metabolite of irinotecan, were able to induce a 50% growth inhibitory effect evaluated at 24 h drug treatment (Figure 7a). Thioridazine seemed to induce an arrest in G_1_ phase of the cell cycle and a decrease in the rate of S phase progression towards G_2_M phases. The active metabolite of irinotecan caused an accumulation of cells in the G_2_M cell cycle phases (Figure 7b). We then investigated the mechanism of cell death induced by these two drugs by using annexinV/PI flow cytometric assay. Both thioridazine and SN-38 caused apoptosis in a 50% of the cells at 24 h of treatment (Figure 7c).

In summary, in this study we provided evidence that trabectedin acts on myelomonocytic cells with a high degree of specificity. First of all, our data indicate the possible central role of the *MAFB* transcription factor given its very high upregulation along the treatment and its dual role as regulator and regulated in the network of transcriptional factors possibly implied in the mechanism of action of the drug. We also showed that trabectedin causes the inhibition of the cell cycle pathway with a blockade of cells at the G1/S phase. This effect is possibly related to upregulation of *CDKN1A* gene whose pleiotropic action is involved also in cell cycle regulation.

Finally, the unexpected high correlation between trabectedin and irinotecan or between trabectedin and thioridazine, although requiring comparative experimental mechanistic data, invites to speculate that there may be potential new rationals to combine these drugs to obtain synergistic antitumor effects.

## Figures and Tables

**Table 1:** First four results of the analysis computed with CMAP.

*Rank*	*Cmap name*	*Mean*	n	*Enrichment*	P	*Specificity*	*Percent non-null*
1	Irinotecan	0.946	3	1	0	0.0364	100
2	Phenoxybenzamine	0.766	4	0.986	0	0.0446	100
3	Resveratrol	0.615	9	0.746	0	0.049	100
4	Thioridazine	0.421	20	0.642	0	0.0731	85

Cmap name, name of the perturbagen; mean, arithmetic mean of the connectivity scores for the related instances; *n*, number of instances connected to the query; Enrichment, enrichment score; P, P-value; Specificity, estimation of the uniqueness of the connectivity between the query signature and a set of instances; Percent non-null, percentage of instances in the set with a positive connectivity-score.
