# Supplementary material for: A systems biology approach to investigate the mechanism of action of trabectedin in a model of myelomonocytic leukemia
Source: Pharmacogenomics J. 2016 Dec 13;18(1):56–63. doi: 10.1038/tpj.2016.76 (PMC5817395; doi:10.1038/tpj.2016.76)
Supplement: Supplementary Information [file tpj201676x1.docx]

**Supplementary Information**


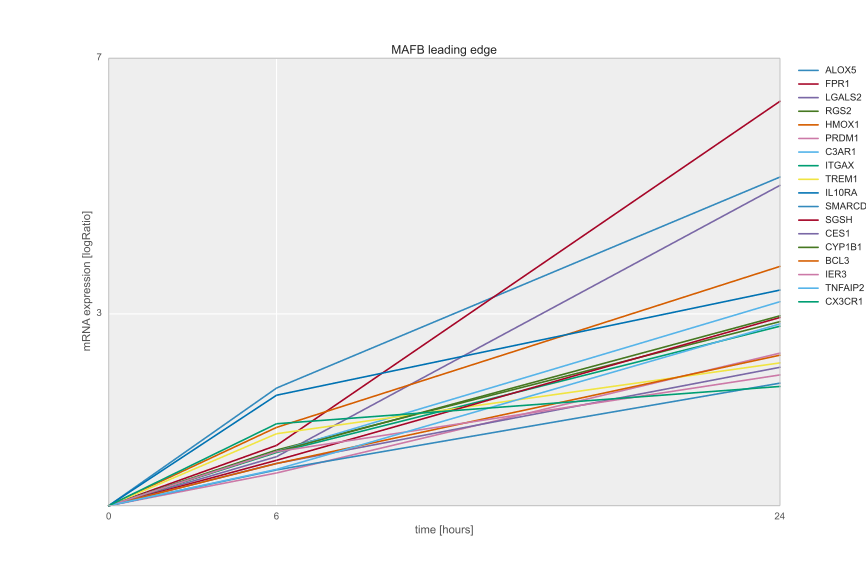
**Supplementary Figure 1**. MAFB enriched leading-edge at 24 hours. Genes show a similar trend in log expression values.

**
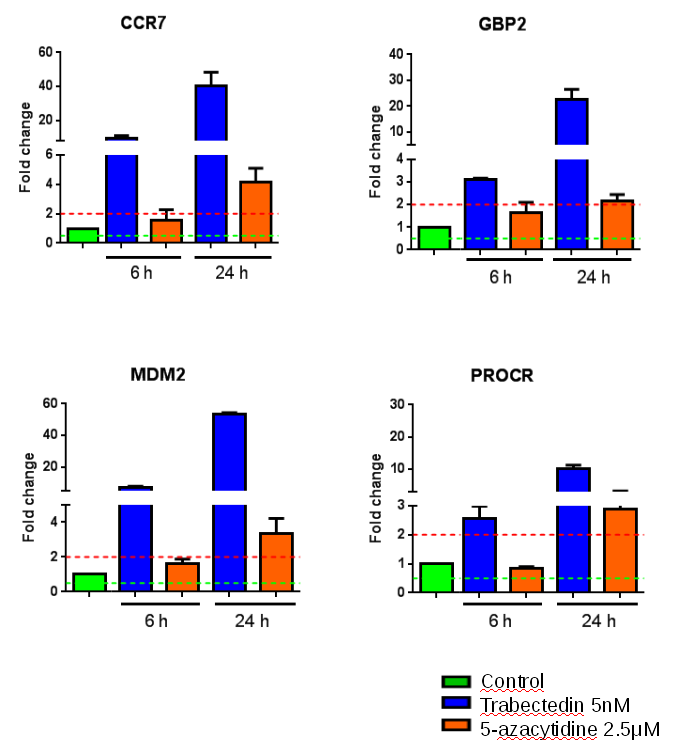
**

**Supplementary Figure 2.** qRT-PCR results for CCR7, GBP2, MDM2 and PROCR genes shared by trabectedin, irinotecan and thioridazine signatures, not differentially expressed with 5-azacytidine. qRT-PCR was performed in triplicates as previously published [1]. Green dashed line: 0.5 fold change; red dashed line: 2 fold change. Data were analyzed by the DDCT method and expressed as fold change (arbitrary unit) compared to untreated control (set as 1).


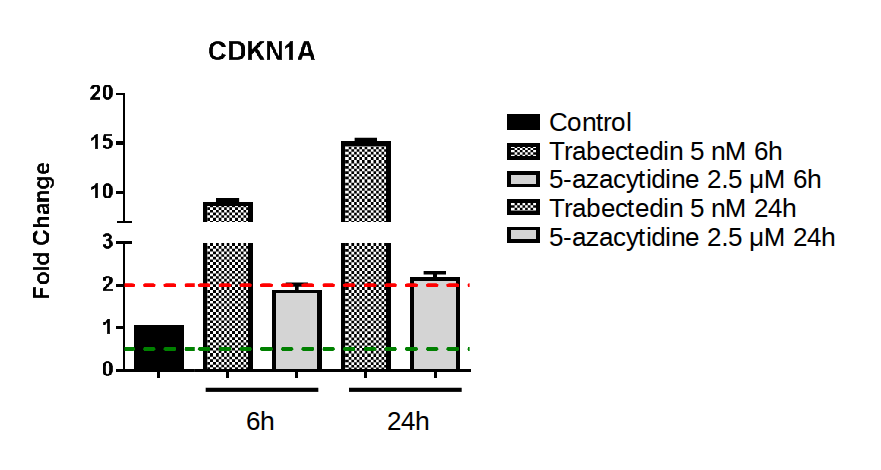


**Supplementary Figure 3**. qRT-PCR results for CDKN1A gene. Control is set to the value of 1 as reference. qRT-PCR was performed in triplicates as previously published [1]. Green dashed line: 0.5 fold change; red dashed line: 2 fold change. Data were analyzed by the DDCT method and expressed as fold change (arbitrary unit) compared to untreated control (set as 1).

**Supplementary Table**

**Supplementary Table 1**. Differentially expressed genes with trabectedin at 6 hours (attached).

**Supplementary Table 2.** Differentially expressed genes with trabectedin at 24 hours (attached).

| **NAME** | **ES** | **FDR q-val** | **NAME** | **ES** | **FDR q-val** |
| --- | --- | --- | --- | --- | --- |
| **6 hours enrichment** | | | | | |
| **NR4A2** | 0,817 | 0,0029 | **FOSL1** | 0,771 | 0,0041 |
| **JUNB** | 0,648 | 0,0014 | **IRF7** | 0,750 | 0,0065 |
| **SOXI** | 0,752 | 0,0023 | **NFKB2** | 0,575 | 0,0102 |
| **NR4AI** | 0,702 | 0,0040 | **OVOL2** | 0,601 | 0,0268 |
| **STAT4** | 0,798 | 0,0032 | **ID2** | 0,663 | 0,0287 |
| **MAFF** | 0,714 | 0,0031 | **ZBTB7B** | 0,574 | 0,0267 |
| **24 hours enrichment** | | | | | |
| **JUNB** | 0,730 | 0,000 | **MYCLI** | 0,450 | 0,004 |
| **NFKB2** | 0,630 | 0,000 | **ZNF318** | 0,470 | 0,004 |
| **NR4AI** | 0,700 | 0,000 | **RELB** | 0,590 | 0,004 |
| **MTFI** | 0,640 | 0,000 | **CAMTA2** | 0,570 | 0,005 |
| **IRF7** | 0,760 | 0,000 | **TFEB** | 0,400 | 0,006 |
| **ZFP36LI** | 0,540 | 0,000 | **ELF4** | 0,650 | 0,007 |
| **CEBPB** | 0,480 | 0,000 | **KLF4** | 0,420 | 0,006 |
| **CREB5** | 0,690 | 0,000 | **FOSLI** | 0,620 | 0,010 |
| **NR4A2** | 0,760 | 0,000 | **PLAGL2** | 0,590 | 0,009 |
| **HIF1A** | 0,620 | 0,000 | **SOXI** | 0,600 | 0,010 |
| **ZBTB7B** | 0,590 | 0,000 | **VDR** | 0,400 | 0,009 |
| **RARA** | 0,510 | 0,000 | **POU2F2** | 0,460 | 0,010 |
| **ID2** | 0,690 | 0,000 | **SATB2** | 0,540 | 0,010 |
| **PRDMI** | 0,570 | 0,000 | **MAF** | 0,660 | 0,010 |
| **FOSL2** | 0,610 | 0,000 | **REL** | 0,580 | 0,011 |
| **BCL6** | 0,460 | 0,000 | **STAT4** | 0,630 | 0,012 |
| **MAFB** | 0,460 | 0,001 | **ATF3** | 0,660 | 0,017 |
| **OVOL2** | 0,610 | 0,001 | **JUND** | 0,490 | 0,017 |
| **TFE3** | 0,590 | 0,002 | **SP11O** | 0,460 | 0,018 |
| **SPII** | 0,440 | 0,002 | **NR4A3** | 0,480 | 0,025 |
| **IRF8** | 0,440 | 0,002 | **CREM** | 0,590 | 0,025 |
| **RXRA** | 0,440 | 0,002 | **IRF5** | 0,390 | 0,026 |
| **ZNF467** | 0,450 | 0,003 | **AHR** | 0,410 | 0,042 |
| **STAT6** | 0,510 | 0,004 | **GRHL2** | 0,400 | 0,043 |
| **MAFF** | 0,600 | 0,004 | **LEF1** | 0,530 | 0,046 |

**Supplementary Table 3.** **Gene-sets from GSEA.** Most significant gene-sets from GSEA at 6 and 24 hours (corrected p-value <0.05).

| **trabectedin gene-sets** | **azacitidine gene-sets** |
| --- | --- |
| ZBTB7B | PKNOX1 |
| CEBPB | ZNF335 |
| PRDM1 | RXRA |
| NFKB2 | MAF |
| OVOL2 | NR4A3 |
| CREB5 | RXRG |
| HIF1A | CDX1 |
| FOSL2 | EZH1 |
| MAFB | PAX8 |
| TFE3 | E4F1 |
| MTF1 | GATA3 |
| ID2 | ZNF839 |
| BCL6 | SIX2 |
| SPI1 | GON4L |
| **gene-sets in common** | |
| RARA | ZFP36L1 |
| JUNB | NR4A1 |
| NR4A2 | IRF7 |

**Supplementary Table 4. Enrichment analysis comparison between trabectedin and 5-azacyditine.** Trabectedin gene-sets: enriched gene-sets from comparison trabectedin versus control at 24 hours. 5-azacytidine gene-sets: enriched gene-sets from comparison 5-azacytidine versus control at 24 hours. Gene-sets in common: genes-sets in common between the enrichment of trabectedin and 5-azacytidine at 24 hours.

**References**

1. Germano G, Frapolli R, Belgiovine C, Anselmo A, Pesce S, Liguori M, et al. *Role of macrophage targeting in the antitumor activity of trabectedin.*, Cancer Cell 2013; 23: 249-262.
